# Development of a quality assurance handbook to improve educational courses in Africa

**DOI:** 10.1186/1478-4491-6-28

**Published:** 2008-12-18

**Authors:** Helen M Nabwera, Sue Purnell, Imelda Bates

**Affiliations:** 1 Liverpool School of Tropical Medicine, University of Liverpool, Liverpool, UK; 2 Educational Development Division, Centre for Lifelong Learning, University of Liverpool, Liverpool, UK

## Abstract

**Background:**

The attainment of the Millennium Development Goals has been hampered by the lack of skilled and well-informed health care workers in many developing countries. The departure of health care workers from developing countries is one of the most important causes. One of the motivations for leaving is that developed countries have well-established health care systems that incorporate continuing medical education, which enables health care workers to develop their skills and knowledge base. This provision is lacking in many developing countries. The provision of higher-education programmes of good quality within developing countries therefore, contributes to building capacity of the health care workforce in these countries.

**Methods:**

The Liverpool School of Tropical Medicine is involved in delivering off-site higher educational programmes to health care workers in Africa. Our colleagues at one of these sites requested a guide to help them ensure that their professional development courses met international educational standards. We reviewed published literature that outlines the principles of quality assurance in higher education from various institutions worldwide. Using this information, we designed a handbook that outlines the quality assurance principles in a simple and practical way. This was intended to enable institutions, even in developing countries, to adapt these principles in accordance with their local resource capacity. We subsequently piloted this handbook at one of the sites in Ghana. The feedback from this aided the development of the handbook. The development of this handbook was participatory in nature.

**Results:**

The handbook addresses six main themes that are the minimum requirements that a higher education course should incorporate to ensure that it meets internationally recognized standards. These include: recruitment and admissions, course design and delivery, student assessments, approval and review processes, support for students and staff training and welfare. It has been piloted in Ghana and the feedback was incorporated into the handbook. The handbook is currently available free of charge online and being used by various institutions across the world. We have had responses from individuals and institutions in Africa, Asia, North America and Europe.

**Conclusion:**

The principles outlined in the handbook provide a regulatory framework for locally establishing higher education courses of good quality that will contribute to enhancing the teaching and learning experience of students in courses in the developing world. This would contribute to providing a skilled and sustainable health care workforce that would reduce the need for health care workers to travel overseas in search of good higher education courses.

## Background

At the United Nations in 2000, world leaders agreed to work towards attaining eight Millennium Development Goals by 2015 that aim to enhance the attainment of security, development and human rights for citizens of all Member States [1]. The three goals that address the health care needs of the world's populations include reducing child mortality, improving maternal health and combating HIV, malaria and other diseases. The attainment of these goals has been hampered by the lack of skilled and well-informed health care workers in many developing countries [1,2]. The reasons for this are multi-factorial, but the departure of health care workers from developing countries is one of the important causes. The International Organization for Migration estimates that since 1990, Africa has continued to lose its skilled personnel at an estimated rate of 20 000 per year [3]. In Africa, the loss of physicians and nurses has been the most striking [4,5]. One of the motivations for this exodus is that developed countries have well-established health care systems that incorporate continuing medical education, which enables health care workers to develop their skills and knowledge base [2,3]. This provision is lacking in many developing countries. Other reasons for emigration include failing economies, political crises, high unemployment rates, human rights abuses and armed conflict [6].

The provision of higher-education programmes of good quality within developing countries is a solution to building the capacity of the health care workforce in these countries [7-9]. Measures of quality of courses are varied and their implementation is labour- and resource-intensive. This therefore requires commitment from governments and other stakeholders involved in setting up training programmes for health care workers. The measures of quality or performance indicators include the qualifications of student entrants and academic staff; resources available; opinions of academic peers; student completion rates; student appraisal of staff; student employability; and employer and staff satisfaction [10]. These measures are important, as they facilitate benchmarking with other institutions running similar courses. Unfortunately, the quality of higher-education courses in African countries is rarely assessed.

In response to the emergence of globalization, decentralization, increased demand for higher education and reduced funding for higher education, governments worldwide (particularly in the developed world) introduced quality assurance measures in their higher-education institutions in order to safeguard the rigour of their awards [8,9,11-13]. In the United Kingdom, Lord Dearing chaired a National Committee of Inquiry into Higher Education that outlined its recommendations in the Dearing report [14]. Following this report, the Quality Assurance Agency for Higher Education Policy for England, Wales and Northern Ireland was formed in 1997. Its role was to ensure that all higher-education institutions in the United Kingdom deliver educational programmes that are quality-assured to a high and clearly defined standard. [15] Some African countries have also appointed specific governing bodies to implement quality assurance in higher education [11]. These tend to be university- and institution-specific and may not address the needs of the wide variety of higher-educational courses on offer.

The Liverpool School of Tropical Medicine is involved in delivering off-site higher-educational programmes to health care workers in Africa. Our colleagues at one of these sites, a teaching hospital in Kumasi, Ghana, West Africa, requested a guide to help them ensure that their professional development courses met international educational standards [16]. It is against this background that we set about designing a handbook that outlines the principles of quality assurance in higher education in a simple and practical way that would enable institutions in developing countries to adapt these principles in accordance with their local resource capacity.

## Methods

The specific needs of the Ghanaian users were identified through discussions with the hospital chief executive officer, medical school dean, medical director, heads of departments, health workers and potential students, and through the personal experience of one of the authors (IB) who lived and taught in Ghana. The process for developing the handbook to meet these needs was devised by the authors in December 2005.

We conducted a literature search, during which we reviewed publications that addressed quality assurance in higher education worldwide in order to incorporate internationally recognized principles into the handbook. From the United Kingdom we reviewed documents from the Quality Assurance Agency and the University of Liverpool's Teaching Quality Support Division web sites. [15,17]. We also reviewed documents that focused on quality assurance strategies internationally, in particular in Africa, from World Bank and African institutional web sites as well as published literature [8-12,18-23]. This information was particularly relevant, as it gave us information on problem-solving strategies that would enable quality assurance principles to be implemented in resource-limited settings.

The aim of this handbook was to provide a short, simple and jargon-free set of principles on quality assurance transferable to any higher-education course. The first draft was completed on 28 February 2006; between February and May 2006 it was reviewed by colleagues at a teaching hospital in Kumasi, Ghana (in the context of courses they were teaching at the time), and by two independent educational experts. This resulted in refinements to the handbook, including further reducing any jargon and altering the wording to ensure that the principles were not institution- or subject-specific and could be applied to any educational initiative from a one-day workshop to a master's degree programme. We also included a glossary to ensure that both students and tutors understood all the terms used. We also made it relevant to resource-poor setting by including relevant case studies from the literature demonstrating quality assurance principles.

## Results and discussion

The handbook addresses six main themes that are the minimum requirements a higher education course should incorporate to ensure that it meets internationally recognized standards. These are summarized below.

### 1. Quality assurance of recruitment and admissions

#### Aim

To ensure that courses are accessible to the entire community and that the admissions procedures are fair, transparent and subject to regular reviews.

We encourage tutors to consider introducing innovative schemes to ensure accessibility to disadvantaged students, including female students, disabled students and those from difficult socioeconomic backgrounds. In Uganda, Makerere University has increased female admissions through a weighted admissions system [18].

### 2. Quality assurance of course design and delivery

#### Aim

To ensure that internationally recognized standards are being achieved and that the courses provide students with knowledge and skills that are relevant to the current job market locally, nationally and internationally.

We advocate that the curriculum design be guided by benchmark statements for specific subjects that may be national or international. The course should also be designed in consultation with students, employers and funders, to ensure relevance to local needs. For example, the course may be designed to address local health priorities guided by the attainment of the Millennium Development Goals. We emphasize the need for tutors to vary their teaching methods, as not all students learn well through lectures, for example. The abolition of tutorials at Makerere University in Uganda was associated with a perceived decline in academic standards and a review recommended the reinstitution of tutorials to ensure the quality of academic programmes [18]. Students should also be trained in generic skills, such as the ability to perform literature searches and to critically appraise published literature. They should also have access to information and communication technology and well-resourced libraries. We recognize that this may be difficult to implement in resource-poor settings, but some institutions have been successful in making this provision through, for example, donor funding or using Internet resources that are free for poor countries [12,18,24].

### 3. Quality assurance of student assessments

#### Aim

To ensure that the intended learning outcomes have been achieved and that the academic standard of each course is maintained.

We highlight the need to ensure that the assessments strategies are valid (i.e. they measure the student's ability to meet the course learning outcomes) and fair (i.e. they do not discriminate against minority students) [19-21]. We also highlight the need to have policies in place that clarify for the students issues of academic honesty and correct referencing of material used in assignments. This would include clearly defined penalties for plagiarism and collusion.

### 4. Quality assurance of approval and review processes

#### Aim

To maintain the academic quality of courses and ensure that courses remain relevant in the light of developing knowledge in the discipline.

We stress the importance of having clear procedures for approval of new courses and modification of existing ones. We also advocate a regular review of courses in order to identify good practice that can be disseminated, as well as areas of weakness that can be addressed and improved on. This should include obtaining feedback from students and from employers, such as local health care departments, that would facilitate the development of good courses that fit their purpose and are relevant to the local needs of the community.

### 5. High-quality support for students

#### Aim

To optimize students' learning experiences and equip them to manage their personal and professional development.

We advocate a range of support services, including health and counselling services, financial and budgeting advice and student learning support. Financial and health difficulties are common among students in higher-education institutions in developing countries, and the outcomes can be devastating. Case studies in a number of African countries in 2001 assessed the causes and effects of HIV/AIDS on university campuses and found that, among other issues, female students were particularly vulnerable, as they were less empowered to abstain from sex or negotiate safe sexual practices due to fear of losing financial support [22].

### 6. Staff training and welfare

#### Aim

To empower staff to fulfil their evolving roles in higher education and ensure the delivery of high-quality programmes

Improving the educational skills of tutors is also vital in the quality assurance of higher-education programmes. This enables tutors to maintain high teaching standards, meet their individual goals and respond to their evolving roles in education. A lecturer who participated in such a course in South Africa made the following comments:

I think it's opened my eyes to the complexity of teaching and lecturing. You know different things like, for example, accommodating cultural diversity and other differences with regard to gender, age, etc. [23]

## Conclusion

This is the first handbook that provides clear guidance on the principles of quality assurance. There are challenges in adapting guidelines from the higher-education sector in wealthy countries to make them generic for all professional development courses in a developing country. These include cultural and social differences as well as limited resources. We overcame these difficulties by pilot-testing the handbook at a medical training institution in Ghana, in addition to having it reviewed by independent experts in higher education. The principles outlined in the handbook help to provide a regulatory framework to guide development and management of higher-education courses of good quality, including professional development courses, that will contribute to enhancing the teaching and learning experience of students in courses in the developing world. The provision of high-quality education in their own country will reduce the need for health care workers to travel overseas in search of internationally recognized higher-education courses, and will contribute to providing a skilled and sustainable workforce. The handbook will enable tutors to evaluate and improve on the quality of their course. It also enables stakeholders to effectively target their funds in order to ensure that the training courses they support are of internationally recognized standards.

This handbook is currently available in the public domain via the Internet . We aim to use the feedback we get from various course providers worldwide to develop it further.

## Competing interests

The authors declare that they have no competing interests.

## Authors' contributions

IB conceived the idea of the handbook and managed the project. HN conducted a detailed review of the literature and produced the first drafts of the handbook and this paper. SP provided case studies and technical advice about educational development. All authors contributed to identifying materials for the handbook and this paper, drafting the handbook and this paper, and have approved the final version of the paper.
